# The impact of low erythrocyte density in human blood on the fitness and energetic reserves of the African malaria vector *Anopheles gambiae*

**DOI:** 10.1186/1475-2875-12-45

**Published:** 2013-02-01

**Authors:** S Noushin Emami, Lisa C Ranford-Cartwright, Heather M Ferguson

**Affiliations:** 1 Institute of Infection, Immunity and Inflammation, College of Medical, Veterinary & Life Sciences, University of Glasgow, Sir Graeme Davies Building, 120 University Place, Glasgow, G12 8TA, UK; 2 Institute of Biodiversity, Animal Health and Comparative Medicine, College of Medical, Veterinary & Life Sciences, University of Glasgow, Graham Kerr Building, University of Glasgow, Glasgow, G12 8QQ, UK

**Keywords:** Malaria, *Anopheles gambiae*, Mosquito vector, Erythrocyte density, Fitness, Energetic reserves

## Abstract

**Background:**

Anaemia is a common health problem in the developing world. This condition is characterized by a reduction in erythrocyte density, primarily from malnutrition and/or infectious diseases such as malaria. As red blood cells are the primary source of protein for haematophagous mosquitoes, any reduction could impede the ability of mosquito vectors to transmit malaria by influencing their fitness or that of the parasites they transmit. The aim of this study was to determine the impact of differences in the density of red blood cells in human blood on malaria vector (*Anopheles gambiae sensu stricto)* fitness. The hypotheses tested are that mosquito vector energetic reserves and fitness are negatively influenced by reductions in the red cell density of host human blood meals commensurate with those expected from severe anaemia.

**Methods:**

Mosquitoes (*An. gambiae s.s.*) were offered blood meals of different packed cell volume (PCV) of human blood consistent with those arising from severe anaemia (15%) and normal PCV (50%). Associations between mosquito energetic reserves (lipid, glucose and glycogen) and fitness measures (reproduction and survival) and blood meal PCV were investigated.

**Results:**

The amount of protein that malaria vectors acquired from blood feeding (indexed by haematin excretion) was significantly reduced at low blood PCV. However, mosquitoes feeding on blood of low PCV had the same oviposition rates as those feeding on blood of normal PCV, and showed an increase in egg production of around 15%. The long-term survival of *An. gambiae s.s* was reduced after feeding on low PCV blood, but PCV had no significant impact on the proportion of mosquitoes surviving through the minimal period required to develop and transmit malaria parasites (estimated as 14 days post-blood feeding). The impact of blood PCV on the energetic reserves of mosquitoes was relatively minor.

**Conclusions:**

These results suggest that feeding on human hosts whose PCV has been depleted due to severe anaemia does not significantly reduce the fitness or transmission potential of malaria vectors, and indicates that mosquitoes may be able exploit resources for reproduction more efficiently from blood of low rather than normal PCV.

## Background

Anaemia is a serious health threat to people in developing countries [1], and in Africa is common in women and children [1]. Poor nutrition, iron deficiency and infectious diseases, such as malaria and helminth infections, increase the likelihood of anaemia [2,3]. Pregnancy can also contribute to anaemia in women, with, for example, 92.6% of pregnant women in Gombe, Nigeria being defined as anaemic as a result of concurrent parasitic infections [1,4]. Anaemia is characterized by a change in the biochemical composition of several haematological factors [5], with the most notable being a marked reduction in the density of red blood cells (erythrocytes). Reductions in erythrocyte density commensurate with anaemia can be measured in terms of packed cell volume (PCV) [1]. For adult African males and females, the normal range of PCV is 40–50% (14–18 g/dl haemoglobin, Hb) and 30–46%, (13–15 g/dl Hb) respectively [6]. PCV and Hb measurements are considered as haematological indicators for classifying the severity of anaemia, with PCVs in the range of 21-30% and Hb 9.5–13 g/dl being considered as mild anaemia, PCVs of 15–20% and Hb 8–9.5 g/dl as moderate anaemia, and <15% PCV and Hb <8 g/dl as severe anaemia [7]. As PCV is a measure of the number of erythrocytes per unit volume of blood, and haemoglobin the amount of protein (in erythrocytes) per unit volume of blood, PCV and Hb levels are generally highly correlated [6].

While anaemia has important consequences for the health of affected people, it may also influence the resource value of their blood to mosquitoes and other haematophagous insects that feed upon them. Many insect vector-borne diseases such as malaria both cause anaemia and occur with greatest frequency in developing countries where other causes of anaemia such as malnutrition are common. Consequently insect disease vectors frequently encounter anaemic hosts, and any change in host blood quality caused by this condition that influences vector fitness could limit their transmission potential. This could arise both through direct impacts on vector fitness and ability to survive through the pathogen’s extrinsic incubation period, or through indirect impacts on the reproductive success of pathogens within their vectors (e.g., due to host energetic limitation). As red blood cells are the primary resource mosquitoes use for egg production [8], a reduction in total intake would be expected to decrease their reproductive output. Blood is also an important resource for mosquito long-term survival [9-12]. As malaria parasites require a relatively long period of development within their mosquito vectors before they can be transmitted to a new host [13,14], any reduction in mosquito survival during this period will have a major impact on parasite transmission potential.

Although the reduced erythrocyte content of low PCV blood may be expected to reduce mosquito resource intake, there are some aspects of their fitness that could potentially be enhanced. Specifically, previous studies have demonstrated that *Anopheles stephensi* mosquitoes were able to imbibe significantly larger blood meals when the PCV of their rodent hosts decreased from normal (59–45%) to intermediate levels (43–44%) as a consequence of infection by the rodent malaria parasite *Plasmodium yoelii nigeriensis*[2]. However, when host PCV fell further to 15–35% as parasitaemia increased, mosquitoes obtained smaller blood meals than those feeding on blood of normal PCV [2]. The hypothesized cause of this curvilinear relationship between PCV and blood intake is blood viscosity, with blood of high PCV being ‘thicker’ and requiring more energy to imbibe [15]. In contrast, the relatively ‘thinner’ consistency of blood with moderate PCV reduction allows mosquitoes to imbibe blood at a faster rate and thus consume a higher total volume within a fixed period of time [2,16]. However, at low PCV values, the advantage of faster blood uptake may be outweighed by lower red cell density, which diminishes mosquito energetic intake.

These trade-offs between the rate of blood intake and its PCV in human blood spanning the range of normal to severe anaemia have not yet been investigated. Additionally in most previous investigations, reductions in host PCV have been created by malaria infections [2,17]; thus it has not been possible to clearly separate the impacts of parasites and those of low PCV on resultant measures of mosquito vector fitness. By directly manipulating host red cell density independently of infection, the experiment described here specifically tested the impact of variation in human host PCV as likely to arise in malaria-endemic settings on the fitness and transmission potential of African malaria vectors. In addition to assessing impacts on mosquito fitness, the association between blood PCV and the key energetic resources that mosquito vectors require for survival and maintenance (glucose, glycogen and lipids [18-21]) were also evaluated. In combination these results allow evaluation of the role of anaemia-induced reductions in human blood PCV on limiting the fitness and transmission potential of malaria vectors. Results are discussed in the context of the potential epidemiological consequences of variation in host haematology, and its role in shaping the outcome of vector-parasite interactions in malaria.

## Methods

### Mosquito rearing and blood feeding

Mosquitoes were obtained from a laboratory colony of *Anopheles gambiae s.s* (Keele line) maintained at the University of Glasgow under standard insectary conditions of 27 ± 1°C, 70% humidity and a 12-hr light: 12-hr dark cycle. Larvae were fed *ad libitum* on fish pellets (Tetra Ltd, UK). Pupae were collected from the insectary stock and moved into a holding cage for emergence. The adults that emerged were fed *ad libitum* on a 5% glucose solution supplemented with 0.05% (w/v) 4-aminobenzoic acid (PABA).

Human blood and serum was obtained from the Glasgow and West of Scotland Blood Transfusion Service. Ethical approval for the supply and use of human blood and serum was obtained from Scottish National Blood Transfusion Service committee for governance of blood and tissue samples for non-therapeutic use. Whole blood from donors of any blood group was provided in Citrate-Phosphate-Dextrose-Adenine (CPD-A) anti-coagulant/preservative. Prior to use, the preservative and any remaining white blood cells were removed by washing the blood three times with incomplete RPMI medium (RPMI 1640 (Gibco) supplemented with 25 mM HEPES buffer and 50 mg/L hypoxanthine (Sigma)). The red blood cells were resuspended in incomplete medium to 50% v/v and then kept refrigerated at +4°C. Fresh blood was obtained on a weekly basis. Stocks of serum (off the clot) were obtained as frozen packs from different donors of blood group AB. Several packs were pooled and heat-inactivated at 56°C for one hour to remove complement before use. Serum was stored at -80°C until use.

Blood of different PCV was prepared by diluting red cells in appropriate volumes of serum. Within an experiment, the same batch of serum was used for all treatments. Washed erythrocytes were centrifuged at 1,500 × g for 5 min to pellet the cells. The supernatant was then removed and the pellet resuspended in human serum to achieve two different PCVs representative of blood from a normal (normal PCV = 40–50%) and severely anaemic human host (low PCV = 15%). The PCV of the prepared blood mixtures was checked prior to mosquito feeding by drawing a 30 μl sample into a 1.15 × 1.55 × 75 mm capillary tube (Hawksley and Sons Ltd, Lancing, Sussex, UK) and centrifuging for 5 min in a haematocrit centrifuge at 3,300 × g. The resultant PCV was calculated as the percentage of the total volume of the capillary tube that consisted of packed red cells rather than serum [22]. Groups of 130 adult female mosquitoes, of three to five days post emergence, were collected and transferred into cylindrical cardboard holding pots (diameter 90 mm × height 110 mm) sealed with netting in preparation for blood feeding. Mosquitoes were held in these pots for a further two days before blood feeding and provided with glucose/PABA solution *ad libitum*. The day before the blood feed, the glucose solution was removed and the mosquitoes were provided with distilled water only to increase their willingness to take a blood meal through the membrane feeder.

Membrane feeding was carried out following established protocols [23]. Glass membrane feeders covered with Goldbeater’s skin (ZH de Groot, Heemraadssingel 255a, 3023CE, Rotterdom, The Netherlands) were attached to base of glass membrane feeders using elastic bands, and the feeders were connected to a circulating water bath at 37°C. A 1–1.5 ml volume of each blood PCV treatment was placed into separate membrane feeders, which was then lowered onto the surface of a holding pot containing pre-starved *An. gambiae s.s.* Mosquitoes were allowed to feed from the membrane feeder for 15–20 min. Two to three hours after the membrane feed, pots were inspected and all unfed mosquitoes were removed and killed by freezing. All blood-fed mosquitoes were transferred into individual 7 ml plastic bijou tubes which were labelled with a unique identifier code to designate their experimental treatment. These mosquitoes were maintained under standard insectary conditions and given access to a 5% glucose solution containing 0.05% PABA solution through a cotton wool pad placed on top of the netting that sealed the top of the tube. Pads were changed every day. This experiment was repeated seven times, with a group of approximately 130 *An. gambiae s.s* fed on normal and low PCV in each replicate.

### Measuring mosquito fitness parameters

The amount of blood (erythrocytes) taken by individual mosquitoes during feeding was estimated by measuring the amount of haematin excreted after blood feeding, which is correlated with the total mass of erythrocytes imbibed [24]. To measure haematin excretion, individual mosquitoes were kept in separate tubes for three days after feeding to allow blood digestion to be completed. After three days, each mosquito was transferred into a new tube (7 ml) bearing the same ID number, and the haematin deposited in their previous tube measured as an estimate of blood meal size following the methodology of Briegel, 1980. In brief, 1 ml of a 1% (w/v) lithium carbonate solution was added to each tube and mixed well to dissolve the haematin. The absorbance of the resultant solution was measured at 405 nm in an ELISA plate reader (Dynex Ltd, MRX Revelation, San Diego, CA, USA), which had been calibrated against a lithium carbonate-only blank. The amount of haematin in the sample was estimated by comparison to a standard curve prepared using porcine haematin (Sigma) at concentrations of 1 to 30 μg/ml. The blood meal size of each sample (mass of haematin) was calculated from the regression equation obtained from the standard curve.

After haematin collection, mosquitoes were transferred into new plastic tubes (7 ml) that contained water to a depth of 1 cm to allow for oviposition. The next day, all tubes were inspected for eggs. If eggs were present, the mosquito was moved into a new tube, and the number of eggs laid counted under a dissecting microscope (fecundity). Oviposition rate was calculated as the proportion of blood-fed mosquitoes that had laid eggs by three days after the blood feed.

Each day after blood feeding, tubes were examined to check whether mosquitoes were alive, and the day of their death was recorded. After death, the body size of mosquitoes was estimated by measuring their wing-length (a standard indicator of body size) [25]. One wing was dissected from the body and placed in a drop of distilled water on a microscope slide. The length of the wing was measured using a digital camera imaging system (Moticam 2300) connected to a dissecting microscope. Wing length was measured as the distance from the axillary incision to the apical margin [26] using precalibrated software (Motic Images Plus, v.2.0).

### Quantifying mosquito energetic reserves

Colorimetric-based biochemical analyses were performed to assess the levels of energetic reserves in mosquitoes fed on blood of different PCV. Lipid abundance in mosquito bodies was estimated using the vanillin test, and glycogen and glucose abundance was estimated from the anthrone test [27]. Mosquito energetic resources were assayed on their natural date of death. The bodies of mosquitoes found dead on each day were stored at -20°C in individual 1.5 ml microfuge tubes.

For analysis, 0.2 ml of sodium sulphate solution (2% concentration) was added to each tube and the mosquito body was homogenized using a non-stick rod (Fisher Scientific, Kontes, 749521-0590). Tissue from individual mosquitoes was washed into new microfuge tubes with 750 μl chloroform-methanol (1:1) solution. Tubes were then centrifuged (5,268 × g, 1 min) and the resulting supernatant transferred into clean 15 ml tubes. The pellets formed during centrifugation were retained for glycogen analysis and the supernatant was used for lipid and sugar estimations. The supernatant was centrifuged again (5,268 × g, 1 min) and the top fraction was used for sugar analysis and bottom fraction was kept for lipid analysis [28,29].

### Analysis of lipid levels

Briefly, for lipid analysis the supernatant fraction was placed in the heating block at 90-100°C to evaporate the solvent completely. Some 0.2 ml of sulphuric acid (95–98% grade, approx 18 M) was added to each tube and re-heated for 10 min; this process converts the unsaturated lipids to water soluble sulphuric acid derivatives [28]. Five ml of vanillin- phosphoric acid reagent (Sigma) was added to each tube, and allowed to cool to room temperature. The absorbance of the resulting solution was measured in an ELISA plate reader (MRX Revelation TC Absorbance Elisa Plate Reader, USA) at 540 nm. Lipid concentrations in mosquito tissue samples were estimated from a standard curve, which was obtained from soybean oil in chloroform made by serial dilution of a stock solution of 1 mg/ml (seven different concentrations prepared: 5, 10, 20, 40, 80, 160, and 320 μg/ml). Linear regression analysis was used to estimate the relationship between the known concentrations of these prepared standard samples and their absorbance at 540 nm, measured in an ELISA plate reader (MRX Revelation TC Absorbance Elisa Plate Reader, USA). The published protocol [27] recommended a wavelength of 490 nm, for which no filter was available. A wavelength of 540 nm was found to be satisfactory after comparison with a vanillin standard curve. In developing standard curves for biochemical analysis, correlation co-efficients (R^2^) of 0.98 or higher between known concentrations of reserves and their predicted value were taken as acceptable.

### Analysis of glucose and glycogen levels

The chloroform-methanol solution was evaporated from mosquito samples for glucose analysis in a heated block at 90–100°C, until the solvent volume was reduced to 0.1–0.2 ml. Three ml of anthrone reagent (Sigma) was added to each tube and mixed. The mixture was reheated for 17 min at 90–100°C and then allowed to cool to room temperature. After cooling, absorbance (range of green colour) was measured at 630 nm in an ELISA plate reader (MRX Revelation TC Absorbance Elisa Plate Reader, USA). The same process was followed for measurement of glycogen from the pellet, except the reheating step was omitted. The procedure for preparing mosquito samples for biochemical analysis was based on a minor modification of previous protocols [28,29]: the volume of anthrone solution was reduced to 3 ml [30].

Sugar and glycogen concentrations in mosquito tissue samples were estimated from the same standard curve, which was obtained from anhydrous glucose solutions made by serial dilution of a stock solution of 1 mg/ml (eight different concentrations prepared: 32.2, 16.1, 8.0, 4.0, 2.0, 1.0, 0.5 and 0.25 μg/ml). Linear regression analysis was used to estimate the relationship between the known concentrations of the prepared standard samples and their absorbance at 630 nm, measured in an ELISA plate reader [MRX Revelation TC Absorbance Elisa Plate Reader, USA]. The published protocol recommended a wavelength of 625 nm for this test but the ELISA reader, which was used in this experiment had only a 630 nm filter. The accuracy of this wavelength (630 nm) was confirmed by checking the original anthrone standard curve [27,29].

### Statistical analysis

The impact of PCV variation on four indicators of *An. gambiae s.s* fitness was measured: blood meal size, fecundity, oviposition rate and survival. In all analyses, blood PCV treatment was investigated as the primary variable of interest, with mosquito wing length fitted as an additional fixed explanatory variable in all cases. Furthermore, the effect of replicate (seven per treatment) was fitted as a random effect. Associations between the continuous variables of blood meal size and fecundity, and the explanatory variables of wing length and PCV were tested using the generalized linear mixed model procedures (GLMM) in the R statistical package (lmer, R statistical software v.2.10.1/2.12.2, [31]).

A maximal statistical model including all fixed and random effects, and the interaction between fixed effects (PCV × wing length) was created. Non-significant terms were sequentially removed following the backward elimination procedure [31] to yield a final model containing only the statistically significant predictors of these traits. A similar approach was adopted to test for statistical differences between the binary response variable of oviposition (yes/no). Oviposition data were evaluated using logistic regression analysis in R (glmer, R statistical software v.2.10.1/2.12.2 [31]).

The impact of host PCV on mosquito survival was analysed in two different ways. First, the Cox proportional hazard model was used to evaluate the impact of blood PCV on mosquito survival over their entire lifetime. In this model, the random effect of ‘replicate’ was incorporated by fitting a frailty function [32]. The final statistically significant model of long-term survival was identified on the basis of backward elimination as described above. Second, the impact of PCV on the proportion of individuals surviving until 14 days after blood feeding, which is the approximate length of time required for malaria parasites to complete their extrinsic rate of development, was tested [13,14]. This ‘transmission-relevant’ survival parameter was analysed by testing for significant differences in the proportion of mosquitoes surviving until day 14 using the generalized linear mixed models procedure in R for binary data as described above (glmer, R statistical software v.2.10.1/2.12.2, [31]).

An analysis was also conducted to assess how mosquito energetic resources at death varied between different blood PCV treatments. Mosquito body size was fitted as an additional explanatory variable in these analyses, with experimental replicate treated as a random effect. The analyses were conducted using the generalized linear mixed effects procedure (lmer) in the R statistical software (v.2.12.2 [31]).

## Results

### Blood meal size

**Figure 1:**
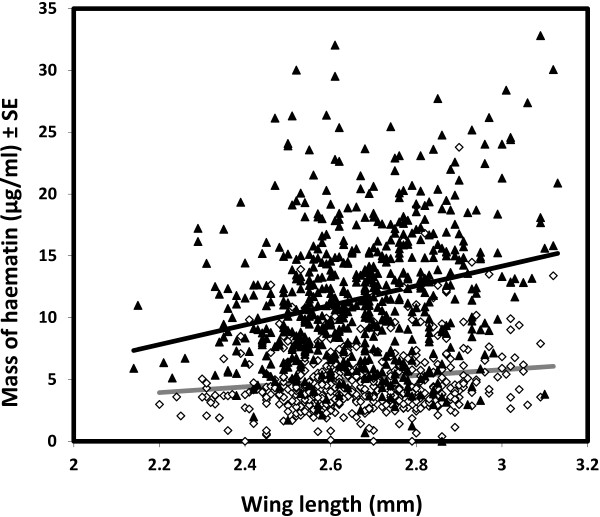
**Relationship between mosquito body size, blood PCV and the size of *Anopheles gambiae *s.s. blood meals.** Each point represents one mosquito. Mosquitoes fed on normal blood are shown as black triangles and those fed on blood of low PCV as white diamonds. The regression lines show statistically significant relationships (normal PCV: solid black line; low PCV: solid grey line).

Approximately 130 female mosquitoes were offered a blood meal of different PCV (50% and 15%) in each of seven replicates (normal PCV: N = 910, low PCV: N = 910, over all replicates). Mosquito blood meal size was significantly influenced by the interaction between blood PCV and mosquito body size (χ^2^_1_ = 13.96, *P* < 0.001, Figure 1). Specifically there was a positive relationship between blood meal size and mosquito body size for both PCV treatments (normal PCV: χ^2^_1_ = 38.18, *P* < 0.001; low PCV: χ^2^_1_ = 19.84, *P* < 0.001), but the rate of increase in blood meal size with mosquito body size was significantly greater in the normal PCV than low PCV treatment (Figure 1). Overall, mosquitoes obtained significantly larger blood meal sizes per unit body size in the normal rather than low PCV group (Figure 1).

### Oviposition and fecundity

**Figure 2:**
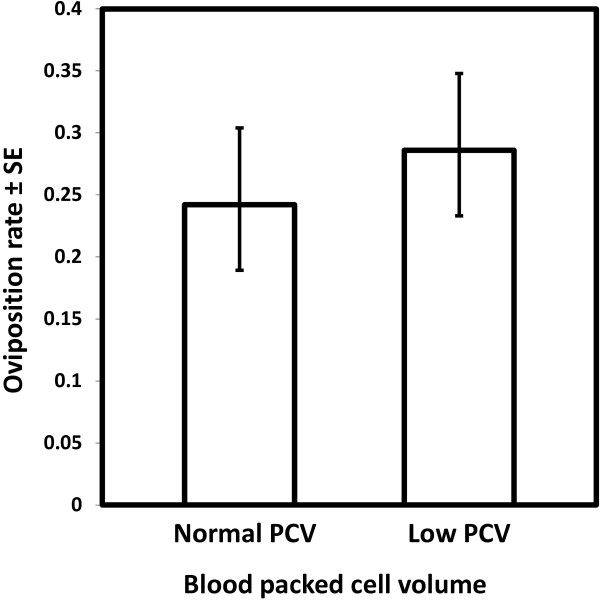
**The oviposition rate of *Anopheles gambiae *s.s. after feeding on human blood of different PCV levels.** Bars show the predicted oviposition rate (proportion of mosquitoes that laid eggs) over seven replicates (N = 1,047). Error bars represent ± 1 standard error (SE).

**Figure 3:**
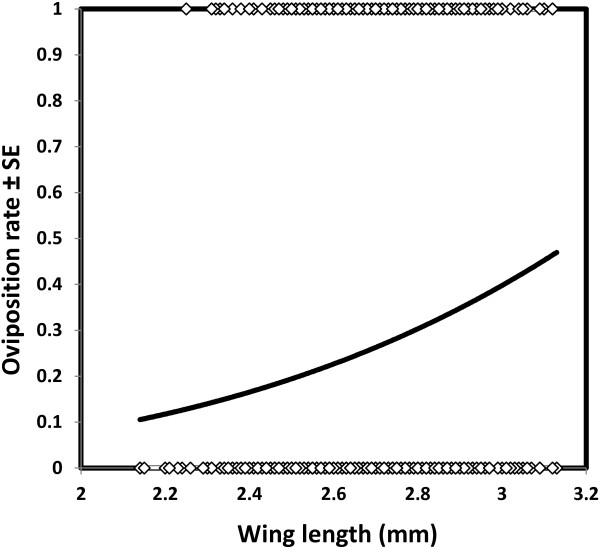
**Predicted relationship between the body size of *Anopheles gambiae *s.s. and their oviposition rate.** Points represent mosquitoes that laid or did not lay eggs (binary response variable).

**Figure 4:**
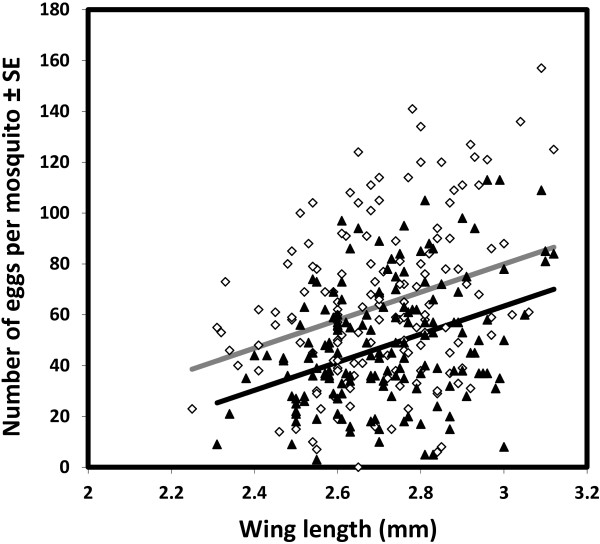
**Relationship between mosquito body size, PCV and the number of eggs laid by ovipositing *Anopheles gambiae *s.s.** Mosquitoes feeding on normal PCV blood are shown as black triangles and those feeding on blood of lower PCV as white diamonds. The regression lines are as predicted by the best-fit statistical model (normal PCV blood: solid black line; low PCV blood: solid grey line).

Although mosquitoes obtained larger meals from blood of normal PCV than from low PCV blood, there was no difference in mosquito oviposition rate after consuming these different blood types (χ^2^_1_ = 2.5, *P* = 0.11, N = 1047, Figure 2). However, mosquito oviposition rate increased with body size (χ^2^_1_ = 22.45, *P* < 0.001, Figure 3). Mosquitoes that failed to lay at least one egg were excluded from further analysis of fecundity. Within those that did oviposit, mosquito fecundity was significantly related to both body size (χ^2^_1_ = 28.50, *P* < 0.001) and blood PCV (χ^2^_1_ = 35.13, *P* < 0.001, Figure 4). For a given body size, mosquitoes feeding on blood of low PCV were predicted to lay approximately 15% more eggs than those who fed on normal blood (Figure 4). There was no significant interaction between PCV and wing length in determining *An. gambiae s.s* fecundity (χ^2^_1_ = 0.6, *P* = 0.44).

### Survival

The survival of all blood-fed mosquitoes was monitored, irrespective of whether they had laid eggs. As reproduction is known to be energetically costly and to reduce longevity in many insects [33-38], the impact of oviposition on *An. gambiae s.s* survival was checked separately to the impact of blood PCV. Mosquitoes that laid eggs were found to have a significantly lower mortality than those that did not (χ^2^_1_ = 15.80, *P* < 0.001, Odds ratio ± 1 SE = 0.72 ± 0.08, 95% CI: 0.61- 0.85, N = 795). All subsequent analyses of the impact of PCV on mosquito longevity were thus performed with the inclusion of oviposition (yes/no) as an additional explanatory variable.

**Figure 5:**
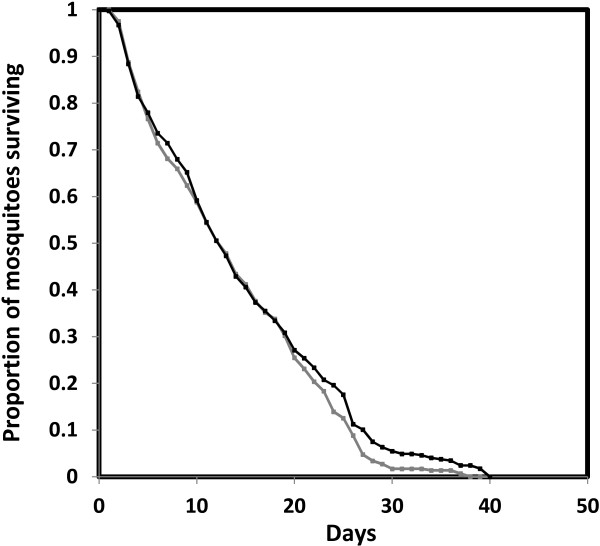
**Survival of *Anopheles gambiae *s.s. female mosquitoes after feeding on blood of different PCV.** Normal PCV indicated by black points and low PCV by grey. Each point was calculated by predicted survival of all replicates and weighting of points depending on variation in the sample size within a replicate. Survival curves were obtained from the Cox proportional hazards model (N = 795).

In this analysis, both oviposition (laid eggs/did not lay eggs, odds ratio of mortality ± 1 SE = 0.70 ± 0.08, 95% CI: 0.59- 0.82, χ^2^_1_ = 14.12, *P* < 0.001) and blood PCV (χ^2^_1_ = 10.99, *P* < 0.001) had a significant impact on mosquito survival. Specifically, the risk of mortality of mosquitoes that fed on blood of normal PCV was approximately 25% lower than in the low PCV group (odds ratio of mortality ± 1 SE = 0.75 ± 0.07, 95% CI: 0.65- 0.87, Figure 5). The long-term survival of mosquitoes in this experiment was unrelated to their body size (χ^2^_1_ = 0.42, *P* = 0.51).

**Figure 6:**
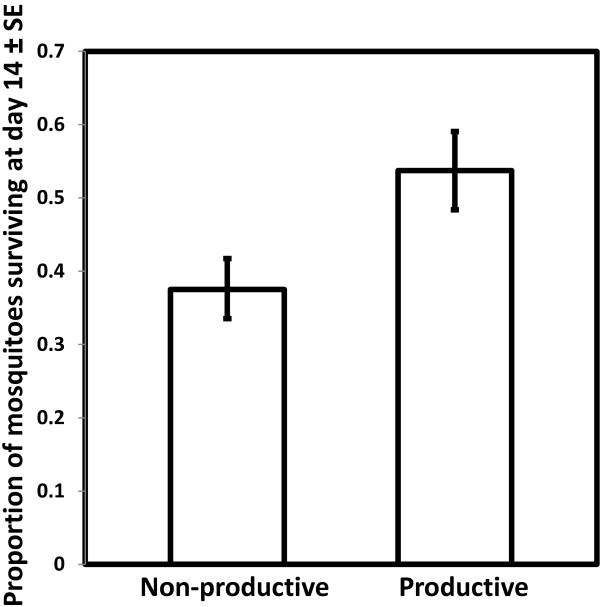
**Predicted proportion of *Anopheles gambiae *s.s. surviving to day 14 after blood feeding.** Mosquitoes are separated in relation to whether they did or did not lay eggs. Error bars represent ± standard error (SE).

Human malaria parasites require approximately 14 days to complete their extrinsic incubation period within mosquitoes before they can be transmitted to a new host [39]. Here, the proportion of mosquitoes that survived for at least 14 days after blood feeding was associated with oviposition rate (χ^2^_1_ = 11.10, *P* < 0.001, Figure 6) but not blood PCV (χ^2^_1_ = 1.71, *P* = 0.19). The survival of mosquitoes until this time was also unrelated to their body size (χ^2^_1_ = 0.91, *P* = 0.34).

### Mosquito energetic resources

**Figure 7:**
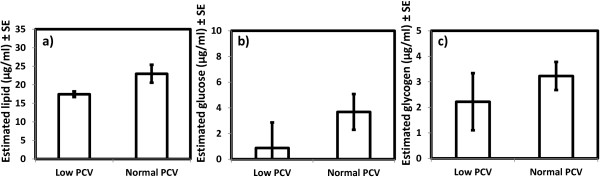
**Mosquito energetic resources after feeding on blood of different PCV.** Energetic resources at death (lipid, glucose and glycogen) in *Anopheles gambiae* s.s. mosquitoes after feeding on blood of different PCV levels (low = 15% and normal PCV = 50%). Error bars represent ± SE. Panels (**a-c**) show levels of lipids (**a**), glucose (**b**) and glycogen (**c**) in mosquitoes fed blood of different PCV (N = 96).

In these experiments, the only significant impact of blood PCV on mosquito energetic reserve levels at death was manifested in lipids (χ^2^_1_ = 4.89, *P* = 0.02, N = 96). Specifically mosquitoes fed on blood of normal PCV had a higher lipid content at death than those fed on blood of low PCV, Figure 7a). There was no significant difference in the abundance of glucose (χ^2^_1_ = 3.68, *P* = 0.055, N = 96) or glycogen reserves (χ^2^_1_ = 1.42, *P* = 0.23, N = 96) in mosquitoes at the time of their death in relation to blood PCV, Figure 7b-c). The abundance of the three reserve types at death was not related to body size (glucose: χ^2^_1_ = 1.90, *P* = 0.17; glycogen: χ^2^_1_ = 3.00, *P* = 0.08; lipid: χ^2^_1_ = 0.49, *P* = 0.48, N = 96).

## Discussion

In this study, reductions in the PCV of human blood of a magnitude consistent with severe anaemia were found to significantly reduce the amount of protein that malaria vectors acquire from blood feeding, and their resultant long-term survival. However mosquitoes feeding on blood of low PCV experienced no reduction in their oviposition rate, and contrary to initial prediction, exhibited increased fecundity of around 15% relative to those fed on blood of normal PCV. Although low PCV blood was associated with a reduction in the long-term survival of mosquitoes, this difference was not evident within the first 14 days after blood feeding, which approximates the minimal time period required for malaria parasites to complete their extrinsic development in mosquitoes. These results suggest that contact with human hosts whose PCV has been depleted due to severe anaemia may not significantly reduce the fitness or transmission potential of malaria vectors.

The observed reduction in blood meal size in mosquitoes feeding on blood of low PCV is consistent with previous studies conducted on rodent models [2,17]. Specifically, anaemia in mice due to infection with *Plasmodium chabaudi* (which reduced their PCV by 20% in comparison to uninfected controls) was associated with a 25% reduction in the blood meal size of *An. stephensi*[17]. A similar study of the rodent malaria parasite *P. yoelii nigeriensis* found that the blood meal size of *An. stephensi* fed on infected mice with low PCV (15–35%) and high parasitaemia was lower than those fed on infected mice with normal PCV (42–45%) [2]. However, as reductions in host PCV in both these studies occurred as a consequence of malaria infection, the impacts of PCV and parasites themselves could not be disentangled. The results on *An. gambiae s.s* presented here demonstrate that PCV variation on its own can account for reductions in mosquito blood meal size that have been associated with feeding on malaria parasite-infected hosts.

The results presented here are in agreement with theoretical predictions that mosquito blood meal size may increase with more moderate levels of PCV reduction [15,16], because reductions in red cell density reduce blood viscosity and thus the rate mosquitoes can imbibe blood. This advantage is predicted to be outweighed by the cost of the lower protein content (red cell density) of blood as its PCV falls [15,16]. Reductions in PCV investigated here were substantial (PCV <15%, consistent with severe anaemia in humans), and resulted in smaller blood meal sizes, confirming the prediction that the advantages of increased blood flow rate with low PCV blood are outweighed by the cost of reduced red cell density at more extreme levels of anaemia (e g, PCV <15%).

In contrast to its influence on mosquito blood meal size, blood PCV had no impact on the oviposition rate of *An. gambiae s.s.*, even after controlling for variation in mosquito wing length which was positively correlated with oviposition rate here and elsewhere [40,41]. It is unclear why extreme reductions in blood PCV that significantly reduced mosquito blood meal size did not reduce their oviposition rate, as in previous studies these factors have been shown to be positively correlated [8,40-42]. Possible reasons could be that the oviposition rate of mosquitoes fed on low PCV blood was enhanced by other chemical substrates in the blood serum such as free amino acids, lipids or other chemical elements. However, currently there is no evidence that blood serum factors might be important in triggering oviposition. Regardless of the mechanism, these results suggest that blood meal size may be an unreliable indicator of the mosquito oviposition rate when females feed from hosts with blood of variable PCV.

Also contrary to expectation on the basis of its impact on blood meal size, blood of low PCV was associated with higher mosquito fecundity. Of mosquitoes that laid at least one egg, those fed on low PCV blood laid approximately 15% more eggs than mosquitoes fed on blood of normal PCV. The finding of enhanced fecundity with low PCV blood is in contrast with previous studies, which have shown that as host PCV decreases due to malaria infection, both mosquito blood meal size and fecundity are reduced [42,43]. The enhanced fecundity of mosquitoes fed on low PCV blood here cannot be due to its higher quality as indexed by protein intake from haemoglobin, as the haematin assay indicated that these blood meals had reduced protein levels. An alternative possibility is that mosquitoes fed on low PCV blood compensated for the lower resource quality of their blood meal by reallocating resources acquired during larval development towards enhanced short-term reproduction, at a cost to long-term survival. This hypothesis is drawn from life-history theory, which predicts that in maximizing their fitness, organisms face a trade-off between the allocation of resources to short-term *versus* long-term reproduction [44-46]. Trade-offs between survival and reproduction are predicted to be most extreme when resource availability is low [44]. Studies on insects such as *Drosophila*, *Ceratitis capitata*, as well as *Aedes* and *Anopheles* mosquitoes, indicate that they can shift resource allocation to short-term reproductive increases, at the expense of longer term survival, when resources are low [47-50]. Further experimentation requires measurement of the total lifetime reproductive success of mosquitoes that are repeatedly fed on low PCV blood meals to confirm whether this apparent enhancement of fecundity is a short-term phenomenon due to resource re-allocation, or a consistent fitness advantage associated with low PCV blood. Such a re-allocation of resources may not completely compensate for the impact of poor blood meal quality (low PCV); eggs laid by mosquitoes fed on low PCV blood may have been provisioned with fewer maternal reserves and consequently have lower hatch rates than those produced from normal PCV hosts (not tested here).

Although variation in blood PCV had a significant impact on mosquito long-term survival, it did not affect *An. gambiae s.s* survival throughout the minimum period required for malaria parasite development (up to 14 days post feeding). This suggests that even extreme variation in host blood PCV may have limited impact on the vectorial capacity of *An. gambiae s.s* mosquitoes. However, although mosquito survival through the parasite’s extrinsic development period is the most crucial determinant of malaria transmission potential, any effect of PCV on mosquito longer term survival beyond this point could have some effect on transmission by reducing the number of biting opportunities mosquitoes have once they become infectious. Consequently, variation in host blood PCV may be expected to have a minor impact on malaria transmission in terms of mosquito long-term survival.

The relatively modest, although mostly statistically significant, differences in mosquito long-term survival associated with blood PCV reported here may be an underestimate of what occurs in the wild, where environmental conditions are harsher and more heterogeneous. Under the standardized insectary conditions used here, mosquitoes were given access to glucose *ad libitum* after blood feeding. Previous studies have shown that sugar feeding enhances mosquito survival [36,47-50]. Thus the provision of glucose to mosquitoes in these experiments may have minimized the fitness costs of low PCV in blood by providing additional energetic resources to fuel survival. Furthermore, under the standardized laboratory conditions used here mosquitoes were exposed to few of the environmental stresses that would be encountered in nature (e.g., temperature and humidity fluctuation, high energetic demand of finding an oviposition site, predators), and thus the cost of resource limitation as experienced in nature may have been underestimated. Furthermore, although not considered in this work, PCV may also influence the frequency of interrupted feeding [16,51]. Field-based experiments considering the impact of human host PCV under natural conditions will thus be required to confirm the role of host haematological factors on malaria vector fitness.

The impact of blood PCV on the energetic reserves of uninfected mosquitoes at death was found to be relatively minor. Lipid abundance at death was higher in mosquitoes fed on normal rather than low PCV blood, but glycogen and glucose levels were similar. Lipid levels are known to be the primary determinant of long-term survival [11], so the greater longevity of mosquitoes fed on normal PCV blood may be explained by this finding. The similarity of glycogen and glucose levels in mosquitoes fed on low and normal PCV blood is perhaps not surprising as these were measured at the time of natural death, when all of these resources may be similarly exhausted as result of general activity, or would have been replenished by *ad libitum* glucose. To more conclusively determine the impact of blood PCV on mosquito energetic reserves, experiments where resource levels are assayed at several points throughout the mosquito life time are needed. However, as reductions in blood PCV had few detrimental impacts on the mosquito fitness traits measured it may also be unlikely to have large impacts on mosquito energetic reserves.

## Conclusions

These results illustrate how variation in the PCV of human blood meals influences the fitness of *An. gambiae s.s*. As expected, the size of blood meals obtained by mosquitoes feeding on blood of low PCV was significantly lower than from blood of normal PCV. However, despite the reduction in blood meal size, mosquitoes that fed on low PCV blood had a similar oviposition rate and produced more eggs than those fed on blood of normal PCV. Feeding on low PCV blood had no impact on the probability that mosquitoes would survive until at least 14 days after blood feeding, the length of time required for malaria parasites to complete their extrinsic development, but did reduce their longer term survival. There are potential implications of these results for malaria transmission. Reductions in the PCV of human blood commensurate with severe anaemia could have some influence on malaria transmission through reduced longer term survival, albeit relatively minor since most mosquitoes would only live long enough for one transmission opportunity anyway [52]. Further investigation of the cumulative impact of repeated feeding on blood of reduced PCV throughout the life time of these mosquitoes is required to confirm the impact of this and other host haematological factors on the fitness and population dynamics of these important malaria vector species.

## Competing interests

The authors declare that they have no competing interests.

## Authors’ contributions

SNE conducted the experiments and drafted the manuscript. LRC and HMF contributed to experimental design and commented on the manuscript. All authors read and approved the final manuscript.
